# Tryptophan metabolism in bipolar disorder

**DOI:** 10.1192/j.eurpsy.2022.310

**Published:** 2022-09-01

**Authors:** F. Fellendorf, M. Platzer, A. Birner, R. Queissner, S. Bengesser, M. Lenger, A. Maget, A. Tmava-Berisha, N. Dalkner, D. Fuchs, J. Gostner, E. Reininghaus

**Affiliations:** 1 Medical University Graz, Psychiatry And Psychotherapeutic Medicine, Graz, Austria; 2 Medical University Innsbruck, Institute Of Medical Biochemistry, Graz, Austria

## Abstract

**Introduction:**

Immune mediated inflammatory processes are involved in the aetiopathogenesis of bipolar disorder (BD) and weight associated comorbidities. Tryptophan breakdown via indoleamine 2,3-dioxygenase-1 (IDO-1) along the kynurenine axis concomitant with a pro-inflammatory state was found more active in BD but also associated with overweight/obesity.

**Objectives:**

Aims of our study were to investigate 1.) the tryptophan metabolism in BD compared to mentally healthy controls, 2.) differences in weight classes, 3.) in a longitudinal setting, dependent on the incidence of BD episodes and euthymia.

**Methods:**

At the Medical University Graz anthropometric and clinical data as well as peripheral tryptophan and kynurenine were assessed in serum samples of 226 individuals with BD and 142 controls. For 75 individuals with BD a longitudinal assessment with three samples was performed. Serum concentrations of tryptophan and kynurenine were determined by reverse-phase high-performance liquid chromatography. The kynurenine/tryptophan was used as a proxy for IDO-1 activity.

**Results:**

showed a higher kynurenine/tryptophan ratio in BD compared to controls and in overweight compared to normal weight persons. Levels remained stable over time. In the longitudinal course, no differences were found between individuals who were constantly euthymic or not as well who had an illness episode or none.

**Conclusions:**

Findings indicate that IDO-1 activity might constitute more a trait and not a state marker of BD. Accelerated tryptophan breakdown along the kynurenine axis may be further facilitated by overweight. This may increase the risk of accumulation of neurotoxic metabolites which impacts BD symptomatology, cognition, and somatic comorbidities.

**Disclosure:**

No significant relationships.

